# Public Opinion About E-Cigarettes on Chinese Social Media: A Combined Study of Text Mining Analysis and Correspondence Analysis

**DOI:** 10.2196/19804

**Published:** 2020-10-14

**Authors:** Di Wang, Joanne Chen Lyu, Xiaoyu Zhao

**Affiliations:** 1 Faculty of Humanities and Arts Macau University of Science and Technology Taipa Macao; 2 Center for Tobacco Control Research and Education University of California, San Francisco CA United States

**Keywords:** e-cigarettes, public opinion, social media, infodemiology, infoveillance, regulation, China

## Abstract

**Background:**

Electronic cigarettes (e-cigarettes) have become increasingly popular. China has accelerated its legislation on e-cigarettes in recent years by issuing two policies to regulate their use: the first on August 26, 2018, and the second on November 1, 2019. Social media provide an efficient platform to access information on the public opinion of e-cigarettes.

**Objective:**

To gain insight into how policies have influenced the reaction of the Chinese public to e-cigarettes, this study aims to understand what the Chinese public say about e-cigarettes and how the focus of discussion might have changed in the context of policy implementation.

**Methods:**

This study uses a combination of text mining and correspondence analysis to content analyze 1160 e-cigarette–related questions and their corresponding answers from Zhihu, China’s largest question-and-answer platform and one of the country’s most trustworthy social media sources. From January 1, 2017, to December 31, 2019, Python was used to text mine the most frequently used words and phrases in public e-cigarette discussions on Zhihu. The correspondence analysis was used to examine the similarities and differences between high-frequency words and phrases across 3 periods (ie, January 1, 2017, to August 27, 2018; August 28, 2018, to October 31, 2019; and November 1, 2019, to January 1, 2020).

**Results:**

The results of the study showed that the consistent themes across time were comparisons with traditional cigarettes, health concerns, and how to choose e-cigarette products. The issuance of government policies on e-cigarettes led to a change in the focus of public discussion. The discussion of e-cigarettes in period 1 mainly focused on the use and experience of e-cigarettes. In period 2, the public’s attention was not only on the substances related to e-cigarettes but also on the smoking cessation functions of e-cigarettes. In period 3, the public shifted their attention to the e-cigarette industry and government policy on the banning of e-cigarette sales to minors.

**Conclusions:**

Social media are an informative source, which can help policy makers and public health professionals understand the public’s concerns over and understanding of e-cigarettes. When there was little regulation, public discussion was greatly influenced by industry claims about e-cigarettes; however, once e-cigarette policies were issued, these policies, to a large extent, set the agenda for public discussion. In addition, media reporting of these policies might have greatly influenced the way e-cigarette policies were discussed. Therefore, monitoring e-cigarette discussions on social media and responding to them in a timely manner will both help improve the public’s e-cigarette literacy and facilitate the implementation of e-cigarette–related policies.

## Introduction

### Background

Electronic cigarettes (e-cigarettes) are devices that look like real cigarettes but deliver a nicotine-containing aerosol to users by heating a solution typically made up of propylene glycol or glycerol (glycerin), nicotine, and flavoring agents [1,2]. As there are fewer carcinogens in the cartridges or vapor of e-cigarettes, they are often marketed as less harmful products or as a healthier alternative to traditional cigarettes, which can help people to quit smoking and even replace the use of traditional cigarettes [3-6]. Studies have also found that e-cigarette use is associated with less negative cardiovascular effects compared with traditional cigarettes, and there may be beneficial effects on blood pressure regulation and endothelial function for smokers who switch to e-cigarettes [7]. Some proponents have called e-cigarettes a revolutionary product in tobacco harm reduction with the potential to significantly reduce the burden of smoking-related diseases globally [8]. In contrast, opponents argue that there has been no conclusive scientific evidence that e-cigarettes are an effective tool for harm reduction compared with smoking combustible cigarettes [2,9] or that e-cigarettes can promote long-term smoking cessation [10]. On the contrary, studies have found that e-cigarette use causes adverse health effects in many organ systems, such as gastrointestinal, cardiovascular, and pulmonary system symptoms [11-14], and even brings changes in the brain that boost the risk of other drug addictions [15]. Besides the health impact, opponents noted that if an increasing number of people think that e-cigarette products are less harmful than cigarettes, this may motivate young people to use e-cigarettes [16,17]. In particular, some e-cigarettes are being promoted as a lifestyle choice and an identity statement [18]. This may make some people who would otherwise not smoke start smoking and serve as a gateway to traditional cigarettes, further prolonging the tobacco epidemic [19]. Therefore, many countries have begun to formulate policies to regulate and control e-cigarettes [20].

The uniqueness of China’s tobacco industry (including e-cigarettes) makes China an especially worthwhile area to study. First, China’s tobacco product market is dominated by the state-owned monopoly of China National Tobacco Corporation and State Tobacco Monopoly Administration (STMA), which are one institution with 2 names [21]. Second, China is the principal producer of e-cigarettes, manufacturing approximately 95% of the world’s e-cigarettes [22]. Although the rate of e-cigarette use in China is still lower than that in many high-income countries, e-cigarettes have become increasingly popular, particularly among young people [23]. In China, e-cigarette sales reached 4.09 billion RMB (approximately US $589 million) in 2017, an increase of 25.3% year-on-year in the consumer market [24]. However, before 2018, China had no regulations on e-cigarettes and their manufacture, distribution, sales, health warnings, packaging, or advertising [25]. The first national-level regulation on e-cigarettes in China was released in August 2018, when China’s State Administration for Market Regulation and STMA jointly issued the *Notice on Prohibiting the Sale of Electronic Cigarettes to Minors*. The notice stated that China’s Minor Protection Law clearly stipulates that it is forbidden to sell tobacco and alcohol to minors and that e-cigarettes, as a supplement to cigarettes and other traditional tobacco products, have big safety and health risks. Therefore, various market entities must not sell e-cigarettes to minors [26]. On November 1, 2019, one more regulation, Notice on Further Protecting Minors from Electronic Cigarettes (Circular No. 1 of 2019), was issued. In addition to stressing the safety risks and health hazards of e-cigarettes, the notice also stipulated that no sales channels are allowed to sell e-cigarettes to minors and that all web-based e-cigarette advertisements should be withdrawn. At the same time, it urged e-cigarette production and sales enterprises and individuals to close e-cigarette sales websites and urged electronic commerce (e-commerce) platforms to take down e-cigarette products and close web-based e-cigarette stores [27]. Therefore, in effect, in China, e-cigarette sales and advertising were banned on the web from November 1, 2019.

### Objectives

On social media, information about e-cigarettes is spreading exponentially among audiences [28]. The public are more likely to rely on information provided by social media, especially when they are uncertain about the long-term consequences of e-cigarettes and where there is a lack of clear policies to regulate e-cigarettes [29]. Therefore, social media have been a popular means for disseminating information [30] and shaping the attitude of the public toward novel health issues [31,32]. Although potential e-cigarette consumers can easily access information on social media, they can also shape discussions around new products [33], which makes social media become both a good platform to gauge public knowledge of health issues [34,35] and an important source for the surveillance of public opinion about e-cigarettes [36-39]. Owing to the accessibility of social media, it is necessary for public health communities and governmental agencies to be aware of information that is being circulated on social media [40]. In particular, after the issuance of policies, social media can provide an efficient platform to access information that may help in understanding how the public perceive the policies; this is critical to regulatory efforts [41]. However, social media studies on public tobacco discussions in China are rare. To the best of our knowledge, there are only 2 relevant studies: one on the nature and extent of e-cigarette discussions on social media in 2013 [42], when China had not yet begun e-cigarette regulation, and one focusing on public reactions to a city-level cigarette control policy in China [43]. Therefore, by examining public conversation about e-cigarettes on social media, this study would be the first to understand what the Chinese public have said about e-cigarettes and how the focus of discussion changed in the context of different policies (ie, in the period before the first e-cigarette regulation in 2018, the period between the first regulation and the issuance of the second in 2019, and the period after the second regulation) to gain insight into how e-cigarette policies have influenced the reaction of the Chinese public to e-cigarettes. To achieve these goals, specifically, this study will identify the most frequently used words and phrases the public used in their discussion of e-cigarettes on Chinese social media and examine the similarities and differences between the high-frequency words the public used in web-based discussion about e-cigarettes across time.

## Methods

### Combining Text Mining and Correspondence Analysis

This study used a combination of the text mining and correspondence analysis to analyze the Chinese public discussion on e-cigarettes. Text mining was used to study the ranking of high-frequency words and phrases during the 3 periods of web-based discussion. To understand the similarities and differences across time in terms of discussion focus, we also conducted the correspondence analysis to examine the relationships between 2 nominal variables, high-frequency words and periods. The results will show the relative positions of various words listed in the frequency table in the form of a perceptual map so that we can see the relationship between high-frequency words and periods. The ability to deal with frequency data provides the correspondence analysis a methodological strength, and the graphical displays provide the correspondence analysis an interpretive strength [44]. In medical research, it has been used to study how the relative frequencies of headache types change with age and the association between personality types and various medical diagnostic groups [45]. It has also been widely used in education [46], tourism [47], and many other fields.

### Data Source Selection

We chose Zhihu as the social media platform to acquire data. *Zhīhū*, which means “Do you know?” in Chinese, is China’s largest question-and-answer platform where questions are asked, answered, and edited by its community of users [48]. It has cultivated a reputation for being one of the country’s most trustworthy social media platforms [49]. Its motto is “Share your knowledge, experiences and thoughts with the world” [50]. As of January 2019, the number of Zhihu users exceeded 220 million and accumulated more than 130 million answers [51]. Unlike Weibo, the most popular microblogging platform in China, on which the posts spreading tobacco control policies were mostly from professional new media accounts [43], the posts about tobacco issues on Zhihu were mostly individual accounts. Thus, we used Zhihu as the platform to study Chinese public discussions about e-cigarettes [48].

### Data Acquisition and Preprocessing

We used Python (Python Software Foundation), a programming language, to retrieve information about e-cigarettes on Zhihu in January 2020. In total, 2 Chinese keywords, “Dian Zi Yan” and “Dian Zi Xiang Yan” (both mean e-cigarette in Chinese), were used to identify all the questions related to e-cigarettes and their corresponding answers through to December 31, 2019, when the study was completed. Owing to the limited number of questions and discussions on e-cigarettes before 2017, our sample selection was from January 1, 2017, to December 31, 2019, with a total of 3275 questions and their answers. Questions about the promotion of a specific e-cigarette brand and questions about how to choose an e-cigarette brand were deleted along with their answers. The removal of advertisements and e-cigarette selection strategies resulted in 1160 questions and their corresponding answers. Next, we used *Jieba* word segmentation software to segment Chinese words and phrases, as there are no spaces in Chinese sentences. In addition, we combined the frequencies of synonyms and filtered out meaningless demonstrative pronouns, conjunctions, and degree adverbs. According to the issue time of China’s 2 e-cigarette policies, we divided the sample into 3 periods. The sample size for the 3 periods was 208 questions and their answers for period 1, 473 questions and their answers for period 2, and 479 questions and their answers for period 3.

## Results

### Text Mining and Analysis

According to the issuing time of China’s 2 e-cigarette policies (August 28, 2018, and November 1, 2019) [26,27], we divided the sample into 3 periods (January 1, 2017, to August 27, 2018; August 28, 2018, to October 31, 2019; and November 1, 2019, to December 31, 2019). 

We first ran the word frequency for the 3 periods separately and listed the top 50 words for each period (Table 1). Among the top 50 frequently mentioned words, “e-cigarette,” “nicotine,” “tobacco tar,” “cigarette,” “smoking cessation,” “smoking,” “harm,” and “tobacco” appeared in all 3 stages. To further our understanding of the relationships between the keywords, we searched the above words in the 1160 questions and found that 2.9% (6/209) of questions in period 1, 10.8% (51/473) of questions in period 2, and 10.5% (50/478) of questions in period 3 were related to the comparisons between e-cigarettes and traditional cigarettes. At the same time, 22.9% (48/209) of questions in period 1, 20.9% (51/473) of questions in period 2, and 18.2% (50/478) of questions in period 3 asked about the harm of e-cigarettes. In addition, 19.6% (41/209) of questions in period 1, 15.6% (74/473) of questions in period 2, and 3.7% (18/478) of questions in period 3 asked about whether e-cigarettes can effectively help smokers quit smoking. Other common words appearing in the 3 stages were e-cigarette–related substances such as “tobacco tar,” “cartridges,” and “atomizer.”

**Table 1:** Top 50 high-frequency words and phrases in different periods.

Rank	Period 1	Period 2	Period 3
1	e-cigarette^a^	e-cigarette	e-cigarette
2	Nicotine	cigarette	cigarette
3	Tar	nicotine	tobacco
4	Cigarette	smoking cessation	harm
5	smoking cessation	smoking	nicotine
6	smoking	harm	smoking
7	Harm	tobacco	country
8	Tobacco	tar	problem
9	Use	problem	channel
10	Smog	health	product
11	Taste	product	industry
12	Health	country	sale
13	Product	use	offline
14	Problem	smoker	brand
15	Smoker	market	market
16	Market	smog	tar
17	Produce	tar	minor
18	Hong Kong	produce	health
19	Addition	tradition	forbid
20	Flavor	America	Cartridges
21	Atomizer	industry	physical store
22	low voltage e-cigarettes	influence	buy
23	Mouthfeel	body	online
24	Tar	burn	smoking cessation
25	Influence	research	smoker
26	Body	low voltage e-cigarettes	supervise
27	Domestic	China	America
28	Personal	brand	tradition
29	Choose	component	China
30	Content	atomize	domestic
31	Research	heat	online
32	Component	content	policy
33	Essence	cartridge	sales prohibition
34	passive smoking	choose	price
35	Tradition	forbid	influence
36	Burn	domestic	e-commerce^b^
37	Brand	formaldehyde	flavor
38	Atomize	company	company
39	Cartridge	addition	use
40	Advice	data	user
41	Equipment	user	enterprise
42	Steam	taste	choose
43	Forbid	supervise	produce
44	Glycerinum	personal	announcement
45	Like	material	protect
46	Recommend	addition	teenager
47	Harmless	friend	advice
48	Country	flavor	develop
49	Price	Hong Kong	authentic
50	Friend	harmless	benefit

^a^e-cigarette: electronic cigarette.

^b^e-commerce: electronic commerce.

In addition to the common ground, the focus of discussion also changed in the 3 periods. In period 1, the public discussions were purely focused on the use of e-cigarettes. For example, *flavor* and *mouthfeel* were discussed more frequently in period 1 than in periods 2 and 3. In period 2, words such as *industry* and *company* were frequently mentioned, which showed that with the release of e-cigarette policy in the second period, relevant departments began to increase supervision and the public began to pay attention to the future development of the e-cigarette industry. Despite these regulations, many people still believe that China’s e-cigarette market has great potential. In the third period, with the release of the notice on protecting minors from e-cigarettes, discussions about the sales of e-cigarettes and the protection of minors became increasingly heated. Words such as *sales*, *policy*, *minors*, and *teenagers* began to appear frequently. Text mining results showed that as the policy changed, the public discussion on e-cigarettes also changed.

It is worth noting that some geographical terms appeared in different periods. For example, *Hong Kong* appeared in the first stage, whereas *China* and *America* appeared in the second and third stages. In period 1, Hong Kong banned the sale of e-cigarettes, which led to the discussion on “How to evaluate the impact of banning e-cigarettes in Hong Kong?” In period 2, the first case of death related to e-cigarette use occurred in the United States, and several states banned the sale of e-cigarettes, leading to the discussion on the impact of these events. Zhihu users often compared e-cigarette policies between China and other places.

In addition, verbs that appeared frequently are also worth noting. Frequently used verbs across the 3 periods were the “use” of e-cigarettes and how to “choose” e-cigarettes. The finding that “use” ranked ninth in the first period, 13th in the second period, and 39th in the third period reflected the declining public interest in discussing the use of e-cigarettes. In addition, there were some verbs that only appeared in the third stage, such as “forbid,” “protect,” and “supervise.” It showed that in the third stage, the public began to pay attention to the meaning of the e-cigarette policies and the measures of the policies.

### Correspondence Analysis

We used SPSS version 25 for the correspondence analysis. The first step is to enter the frequency (the number of times a word is used in the period/total number of words used in the period) of words and phrases in each period as a contingency table, which is a two-way table with the 50 high-frequency words and phrases as row headings and the 3 periods as column headings. Table 2 is a contingency table.

**Table 2:** The 3×50 contingency table.

Words and phrases	Period 1 frequency, n (%)	Period 2 frequency, n (%)	Period 3 frequency, n (%)
e-cigarette^a^	3308 (4.30)	13015 (4.96)	6266 (5.65)
cigarette	821 (1.07)	4101 (1.56)	1449 (1.31)
nicotine	1185 (1.54)	2933 (1.12)	740 (0.67)
harm	693 (0.90)	2942 (1.12)	772 (0.70)
smoking	803 (1.04)	2532 (0.96)	701 (0.63)
smoking cessation	812 (1.05)	2692 (1.03)	348 (0.31)
tobacco	460 (0.60)	1837 (0.70)	1098 (0.99)
tar	912 (1.18)	1466 (0.56)	575 (0.52)
problem	292 (0.38)	1181 (0.45)	626 (0.56)
product	297 (0.39)	1017 (0.39)	618 (0.56)
health	298 (0.39)	1119 (0.43)	466 (0.42)
country	116 (0.15)	864 (0.33)	690 (0.62)
market	198 (0.26)	711 (0.27)	576 (0.52)
use	352 (0.46)	861 (0.33)	232 (0.21)
smoker	201 (0.26)	801 (0.31)	327 (0.29)
taste	344 (0.45)	656 (0.25)	255 (0.23)
industry	0 (0.00)	569 (0.22)	616 (0.56)
brand	136 (0.18)	491 (0.19)	594 (0.54)
smog	348 (0.45)	665 (0.25)	0 (0.00)
tradition	143 (0.19)	621 (0.24)	301 (0.27)
America	0 (0.00)	616 (0.23)	303 (0.27)
forbid	124 (0.16)	438 (0.17)	457 (0.41)
cartridge	130 (0.17)	458 (0.17)	402 (0.36)
tar	167 (0.22)	654 (0.25)	0 (0.00)
influence	167 (0.22)	537 (0.20)	246 (0.22)
sale	0 (0.00)	0 (0.00)	601 (0.54)
produce	193 (0.25)	633 (0.24)	193 (0.17)
China	0 (0.00)	515 (0.20)	294 (0.27)
low voltage e-cigarette	172 (0.22)	519 (0.20)	0 (0.00)
body	166 (0.22)	532 (0.20)	0 (0.00)
choice	162 (0.21)	444 (0.17)	201 (0.18)
channel	0 (0.00)	0 (0.00)	624 (0.56)
minor	0 (0.00)	0 (0.00)	562 (0.51)
component	150 (0.19)	489 (0.19)	0 (0.00)
content	159 (0.21)	461 (0.18)	0 (0.00)
burn	137 (0.18)	527 (0.20)	0 (0.00)
research	153 (0.20)	523 (0.20)	0 (0.00)
flavor	176 (0.23)	326 (0.12)	241 (0.22)
atomize	133 (0.17)	479 (0.18)	0 (0.00)
company	0 (0.00)	408 (0.16)	238 (0.21)
supervise	0 (0.00)	343 (0.13)	325 (0.29)
user	0 (0.00)	371 (0.14)	235 (0.21)
addiction	0 (0.00)	406 (0.15)	0 (0.00)
heat	0 (0.00)	465 (0.18)	0 (0.00)
offline	0 (0.00)	0 (0.00)	600 (0.54)
advice	129 (0.17)	0 (0.00)	175 (0.16)
friends	110 (0.14)	327 (0.12)	0 (0.00)
data	0 (0.00)	387 (0.15)	0 (0.00)
atomizer	172 (0.22)	0 (0.00)	0 (0.00)
teenager	0 (0.00)	0 (0.00)	177 (0.16)
sum	76,976 (14.32)	26,2491 (19.8)	11,0895 (20.85)

^a^e-cigarette: electronic cigarette.

On the basis of the number of categories (k) in the columns of the contingency table, the correspondence analysis extracts k−1 latent variables, also called *dimensions* [52]. In our study, the correspondence analysis of e-cigarette discussions in the 3 periods showed a two-dimensional solution (Table 3). Similar to the principal component analysis, the first dimension explains as much variance as possible, and the second dimension is orthogonal to the first and explains as much of the remaining variance as possible [53]. A chi-square test revealed a value of 0.1 (*df*=98), with a *P* value of .03, which rejects the null hypothesis of no association between the 2 dimensions.

**Table 3:** Summary table of the correspondence analysis.

Dimension	Singular value	Inertia	Chi-square (*df*)	*P* value	Proportion of inertia	
					Accounted for	Cumulative
1	0.428	0.183	N/A^a^	N/A	0.817	0.817
2	0.203	0.041	N/A	N/A	0.183	1.000
Total	N/A	0.224	0.1 (98)	.03	1.000	1.000

^a^N/A: not applicable.

The singular values can be viewed as the correlation between the rows and columns of the contingency table and are similar to the Pearson correlation coefficient in correlation analysis [53]. They should be greater than 0.2 to be accepted as feasible dimensions [54]. Inertia is an indicator of how much of the variation in the original data is retained in the dimensional solution [55]. The singular value and the inertia are directly related, inertia=singular value^2^ [44]. For example, the inertia of dimension 1 is 0.428 × 0.428 = 0.183, which means that the first dimension accounts for 18.3% of the total variability. The second dimension accounts for 4.1% of the total variability, and the total inertia for the whole solution is 2.24%.

The cumulative column shows the proportion of the inertia accounted for by the latent variable. In our case, the ﬁrst dimension accounts for 81.7% of the total variability, and the 2 dimensions account for 100% of the total variability.

**Figure 1:**
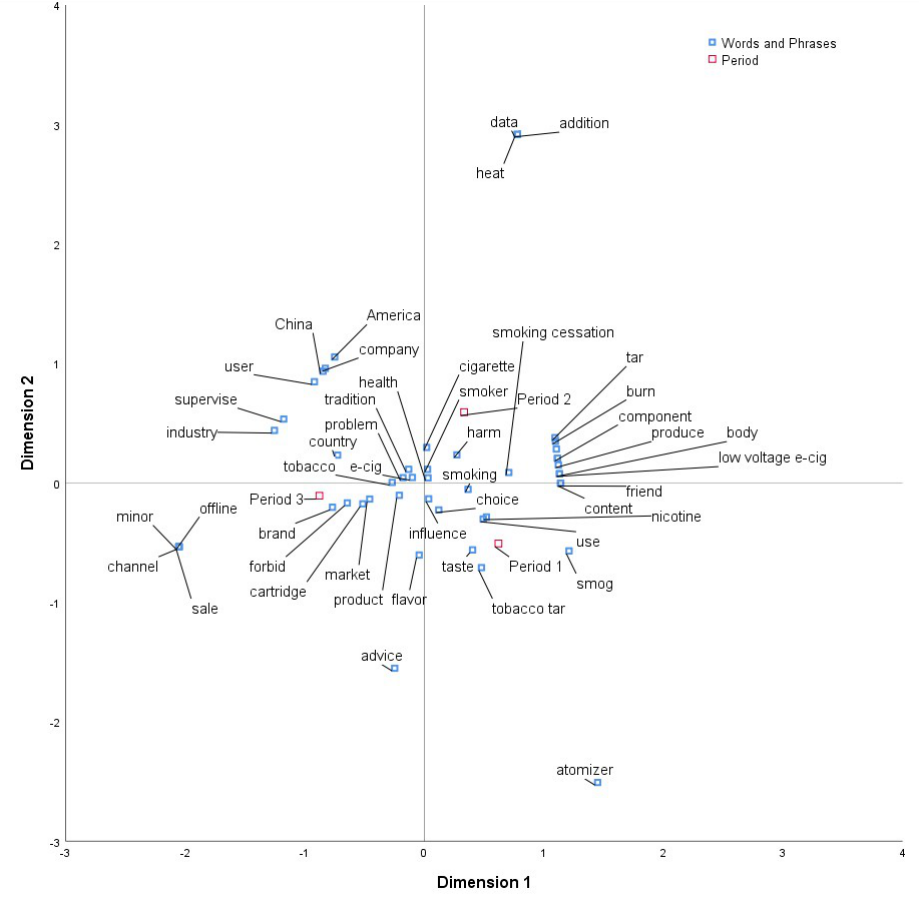
Correspondence map for frequently used words and phrases represented in different periods.

Figure 1 shows the correspondence analysis map that displayed 2 sets of variables, *time periods* and *words and phrases*. The distribution of words was relatively concentrated, and most of them were close to the origin. The closer the words are to the origin, the less distinct they are among the 3 periods. In other words, these words can be considered as the common ground for public discussion of e-cigarettes in the 3 periods. Among them, “e-cigarette,” “health,” “smoker,” “problem,” “tradition,” “influence,” “choice,” “product,” “tobacco,” “harm,” and “smoking” were closed to the origin, which showed that they appeared in similar frequency in the 3 periods.

Specifically, “e-cigarette,” “tradition,” “tobacco,” and “smoking” were similarly represented in the 3 periods. Combining the results from content analysis, we can see that the comparison between e-cigarettes and traditional cigarettes was a common topic. Meanwhile, “health,” “problem,” “influence,” and “harm” were common in all 3 periods. Combing the results from the content analysis, it showed that the public were continually concerned about how e-cigarettes can harm health. In addition, “choice” and “product” appeared with similar frequency in the 3 periods, which showed that the discussion of how to choose e-cigarette products was another common topic across time.

In addition to these similarities, the spots representing the 3 periods appear in 3 completely different directions, revealing differences in the public’s concerns about e-cigarettes in different periods. The further words are from the origin, the more discriminating they are [56]. To interpret the relationship between row and column labels, we need to examine (1) the length of the line connecting the row label to the origin, with longer lines indicating that the association between the row label and some of the column labels is high; (2) the length of the label connecting the column label to the origin, with longer lines again showing that the association between the column label and some of the row labels is high; and (3) the angle formed between the above mentioned 2 lines, with smaller angles indicating that there is a stronger association between the two, a 90° angle indicating no relationship and angles near 180° indicating a negative association [57]. In our case, the row label is *words and phrases*, and the column label is *period.* If a word or phrase meet the abovementioned 3 standards, that is, the length between row label *period* and the origin is relatively long, the length between that word or phrase is relatively long, and the angle between the 2 lines is relatively small, we can say that the word or phrase is a distinct word or phrase that characterizes that period.

Period 1 appeared in the fourth quadrant. Words that met the abovementioned 3 standards were “atomizer,” “tobacco tar,” “smog,” “taste,” “use,” and “nicotine.” Namely, period 1 was characterized by words that reflect elements related to the “use” of e-cigarettes. The public in period 1 concentrated their attention on the identified substances found in e-cigarettes and focused on their feelings of using e-cigarettes. Period 2 appeared in the first quadrant. Words such as “addition,” “heat,” “data,” “tar,” “burn,” “smoking cessation,” and “America” met the abovementioned 3 standards, which showed that in period 2, the public’s attention was not only on the identified substances of e-cigarettes but also on the smoking cessation functions of e-cigarettes. Period 3 appeared in the third quadrant. Words that met the abovementioned 3 standards were “channel,” “offline,” “sale,” “minor,” “brand,” “forbid,” “cartridge,” “market,” “industry,” and “supervise.” This showed that the public paid more attention to the e-cigarette industry and government policy on banning the sale of e-cigarettes to minors at this stage, which was quite different from the previous 2 periods.

## Discussion

### Principal Findings

The rapid popularity of e-cigarettes and the acceleration of regulation has not only attracted media attention [58] but has also generated more discussion on social media. This study used a text mining approach to identify what the Chinese public opinion about e-cigarettes on Zhihu, a social media platform featuring rich discussion. To gain insight into how e-cigarette policies have influenced the reaction of the Chinese public to e-cigarettes, this study further explored the constant focus of public discussion and how this focus changed in the context of different e-cigarette regulations.

The analysis revealed that when talking about e-cigarettes, the consistent themes were comparisons with traditional cigarettes, health concerns, and how to choose e-cigarette products. This may be closely related to the fact that e-cigarettes were marketed as “healthy alternatives to traditional cigarettes” and “the gospel of smokers” when they entered the Chinese market [22]. Although these claims have changed from earlier assertions to open questions in recent years, the Chinese public still associate e-cigarettes with these claims. Although the high-frequency words used in public discussion in the 3 periods had something in common, the differences were greater. The analyses indicated that the issuance of government policies on e-cigarettes led to public discussion on e-cigarettes. Before the first e-cigarette regulation was released in 2018, the discussion of e-cigarettes mainly revolved around the use and experience of e-cigarettes and what e-cigarettes were. Until this period, although e-cigarettes had been available on the market in China for many years (largely because of the lack of regulations or marketing restrictions on e-cigarettes), public understanding and discussion of e-cigarettes was mainly based on industry claims, such as helping to quit smoking, not containing tar, and providing a similar smoking experience to traditional cigarettes but being less harmful than them [22]. In addition, it is worth noting that many discussions in this period came from e-cigarette users. When Zhihu users raised questions about e-cigarettes, these e-cigarette users actively shared their own feelings and experiences and facilitated public conversation.

The second period (ie, the time between the first release of a ban on the sale of e-cigarettes to minors until the second and more comprehensive e-cigarette ban was issued) had many similarities with the first period. For example, many people still focused on e-cigarettes per se in their discussion. Common topics included whether e-cigarettes were an effective smoking cessation tool. However, the promulgation of the *Notice on Prohibiting the Sale of Electronic Cigarettes to Minors* routed the public’s attention to the possible negative effects of e-cigarettes on health, and although “harm” was still a hot topic in this period as in the first period, the core of the harm discussion shifted from the possible harm reduction effect of e-cigarettes in the first period to the possible harm caused by e-cigarettes in the second period. Furthermore, public discussions on e-cigarettes at this stage were more in depth. The notice *triggered*’ discussion on an important issue, the regulation of e-cigarettes by the government. Although the first commercially successful e-cigarette was widely considered to be invented by Chinese pharmacist Hon Lik and China is now the largest producer of e-cigarettes [22], e-cigarettes have always lacked a clear definition in China. According to the Law of the People’s Republic of China on Tobacco Monopoly, e-cigarettes are not tobacco products; they were not in the regulatory scope of either tobacco monopoly law or the state Food and Drug Administration [59]. As stationery stores near primary and secondary schools were reportedly selling e-cigarettes to students [60], the public thought that it was time to regulate the e-cigarette industry, especially for the young vulnerable population. Meanwhile, they began to reflect on why the e-cigarette legislation was lagging. Some people attributed it to the state-owned monopoly of the tobacco industry and the government’s interests from gigantic tobacco taxation [61], including e-cigarette taxation. In this period, the United States also progressed to implementing laws to regulate e-cigarettes, and in July 2019, it launched a new policy requiring an application for deemed *new* tobacco products that were on the market, including e-cigarettes, to be submitted to the Food and Drug Administration [62]. Consequently, the United States easily became a reference that was frequently mentioned when the Chinese public began to talk about the legislation of e-cigarettes in China.

Although there were differences in the focus of discussion among the 3 periods, the biggest difference was between the third period and the former 2 periods. The discussion in the third period jumped far beyond e-cigarettes as a product in itself but put it in a larger social context. In addition to regulatory issues, public discussion also focused on the market, sales, distribution, and other issues regarding the e-cigarette business and industry. This was consistent with the content of the notice and media attention to the policy at that time. The release of the Notice on Further Protecting Minors from Electronic Cigarettes brought forward strict law enforcement, which required various market players to stop selling e-cigarettes to minors and to remove e-cigarette advertisements on the web, and urged e-commerce platforms to close e-cigarette sales channels. In addition, the focus of discussion in this period is very likely connected with mass media reports at that time. A recent analysis of e-cigarette reporting in Chinese newspapers found that an increasing number of news articles referenced policy to frame their reporting in recent years [58]. The findings of this study echoed this analysis, confirming the positive correlation between news coverage on particular issues and public concern about the issues, as found in previous studies [63,64]. A large volume of media reports on the new policies meant that e-cigarettes were no longer just a topic that only e-cigarette users or those who were curious about e-cigarettes had an interest in but a social issue attracting attention from the general public and examined through multiple perspectives. Although the then newly released policy set the agenda for public discussion at that time, media reporting might also have increased the influence of the policy on what the public said about e-cigarettes on social media.

### Limitations

First, it should be cautious to generalize the findings of this study to the whole population of China. On Zhihu, the users basically *speak* as ordinary netizens not opinion leaders [65], which can facilitate public expression. However, as on any social media platform, what we can see on Zhihu are the discussions of those who would like to express themselves on the web; therefore, their viewpoints cannot fully represent those who keep silent and invisible on social media. Second, the focus of this study is high-frequency words in public discussion; therefore, we did not measure emotion, attitude, or opinions. To fully understand the public’s reaction to e-cigarette regulation, future studies that examine the emotional and attitudinal dimensions of public discussion are highly recommended.

### Conclusions

Social media provide an accessible and informative platform to help policy makers and public health professionals understand the public’s concerns over and understanding of health issues, especially the issue of e-cigarettes, where the long-term impacts on health are still uncertain and legislation is still in the early stages. Awareness and monitoring of discussions relevant to e-cigarettes on social media in a timely manner is conducive to the identification of areas where policies need to regulate but have not yet regulated. Meanwhile, if public misunderstandings can be discovered from web-based discussion and guidance and education can be conducted quickly through media or education campaigns, this could help improve the public’s e-cigarette literacy and facilitate the implementation of e-cigarette–related policies.

## Abbreviations

e-cigarette: electronic cigarette
e-commerce: electronic commerce.
STMA: State Tobacco Monopoly Administration

## References

[1] Etter J, Bullen C, Flouris AD, Laugesen M, Eissenberg T (2011). Electronic nicotine delivery systems: a research agenda. Tob Control.

[2] Grana R, Benowitz N, Glantz SA (2014). E-cigarettes. Circulation.

[3] Cahn Z, Siegel M (2011). Electronic cigarettes as a harm reduction strategy for tobacco control: a step forward or a repeat of past mistakes?. J Public Health Policy.

[4] Yao T, Jiang N, Grana R, Ling PM, Glantz SA (2016). A content analysis of electronic cigarette manufacturer websites in China. Tob Control.

[5] Cobb NK, Byron MJ, Abrams DB, Shields PG (2010). Novel nicotine delivery systems and public health: the rise of the 'e-cigarette'. Am J Public Health.

[6] Carr ER (2014). E-cigarettes: facts, perceptions, and marketing messages. Clin J Oncol Nurs.

[7] Kuntic M, Hahad O, Daiber A, Münzel T (2020). Could e-cigarette vaping contribute to heart disease?. Expert Rev Respir Med.

[8] Farsalinos KE, Polosa R (2014). Safety evaluation and risk assessment of electronic cigarettes as tobacco cigarette substitutes: a systematic review. Ther Adv Drug Saf.

[9] Leventhal AM, Strong DR, Kirkpatrick MG, Unger JB, Sussman S, Riggs NR, Stone MD, Khoddam R, Samet JM, Audrain-McGovern J (2015). Association of electronic cigarette use with initiation of combustible tobacco product smoking in early adolescence. J Am Med Assoc.

[10] (2018). Marketers of Electronic Cigarettes Should Halt Unproved Therapy Claims. World Health Organization.

[11] Gaur S, Agnihotri R (2019). Health effects of trace metals in electronic cigarette aerosols-a systematic review. Biol Trace Elem Res.

[12] Pisinger C, Døssing M (2020). A systematic review of health effects of electronic cigarettes. Preventive medicine.

[13] Orellana-Barrios MA, Payne D, Mulkey Z, Nugent K (2015). Electronic cigarettes—a narrative review for clinicians. Am J Med.

[14] Layden JE, Ghinai I, Pray I, Kimball A, Layer M, Tenforde MW, Navon L, Hoots B, Salvatore PP, Elderbrook M, Haupt T, Kanne J, Patel MT, Saathoff-Huber L, King BA, Schier JG, Mikosz CA, Meiman J (2020). Pulmonary illness related to e-cigarette use in Illinois and Wisconsin - final report. N Engl J Med.

[15] Kandel ER, Kandel DB (2014). A molecular basis for nicotine as a gateway drug. N Engl J Med.

[16] Barrington-Trimis JL, Berhane K, Unger JB, Cruz TB, Huh J, Leventhal AM, Urman R, Wang K, Howland S, Gilreath TD, Chou C, Pentz MA, McConnell R (2015). Psychosocial factors associated with adolescent electronic cigarette and cigarette use. Pediatrics.

[17] Pepper JK, Brewer NT (2014). Electronic nicotine delivery system (electronic cigarette) awareness, use, reactions and beliefs: a systematic review. Tob Control.

[18] de Andrade M, Hastings G, Angus K (2013). Promotion of electronic cigarettes: tobacco marketing reinvented?. Br Med J.

[19] Cobb NK, Abrams DB (2011). E-cigarette or drug-delivery device? Regulating novel nicotine products. N Engl J Med.

[20] (2020). Vaporizers, e-cigarettes, and other Electronic Nicotine Delivery Systems (ENDS). US Food and Drug Association.

[21] He P, Takeuchi T, Yano E (2013). An overview of the China national tobacco corporation and state tobacco monopoly administration. Environ Health Prev Med.

[22] Eriksen MP, Mackay J, Ross H (2012). The Tobacco Atlas.

[23] Hennelly W (2015). Market Heats Up for E-cigarettes, Largely Produced in China, as US Scrutiny Rises. China Daily.

[24] Li L, Zhou NB, Qu XH (2018). Market Development and Legal Supervision of New Tobacco Products. China Tobacco Journal.

[25] Juan S (2015). China Plans to Implement E-cigarette Regulations. China Daily.

[26] (2018). Notice on Prohibiting the Sale of Electronic Cigarettes to Minors. State Administration for Market Regulation (SAMR).

[27] (2019). Notice on Further Protecting Minors from Electronic Cigarettes. State Administration for Market Regulation (SAMR).

[28] Gao J, Chapman S, Sun S, Fu H, Zheng P (2012). The growth in newspaper coverage of tobacco control in China, 2000-2010. BMC Public Health.

[29] Lazard AJ, Wilcox GB, Tuttle HM, Glowacki EM, Pikowski J (2017). Public reactions to e-cigarette regulations on Twitter: a text mining analysis. Tob Control.

[30] Emery SL, Vera L, Huang J, Szczypka G (2014). Wanna know about vaping? Patterns of message exposure, seeking and sharing information about e-cigarettes across media platforms. Tob Control.

[31] Cassa C, Chunara R, Mandl K, Brownstein JS (2013). Twitter as a sentinel in emergency situations: lessons from the Boston marathon explosions. PLoS Curr.

[32] Scanfeld D, Scanfeld V, Larson EL (2010). Dissemination of health information through social networks: twitter and antibiotics. Am J Infect Control.

[33] Cole-Lewis H, Pugatch J, Sanders A, Varghese A, Posada S, Yun C, Schwarz M, Augustson E (2015). Social listening: a content analysis of e-cigarette discussions on Twitter. J Med Internet Res.

[34] Lachlan KA, Spence PR, Lin X (2014). Expressions of risk awareness and concern through Twitter: on the utility of using the medium as an indication of audience needs. Comput Hum Behav.

[35] Lazard AJ, Scheinfeld E, Bernhardt JM, Wilcox GB, Suran M (2015). Detecting themes of public concern: a text mining analysis of the Centers for Disease Control and Prevention's Ebola live Twitter chat. Am J Infect Control.

[36] Aphinyanaphongs Y, Lulejian A, Brown DP, Bonneau RP (2016). Text Classification for Automatic Detection of E-cigarette Use and Use for Smoking Cessation From Twitter: A Feasibility Pilot. Proceedings of the Pacific Symposium.

[37] Cole-Lewis H, Varghese A, Sanders A, Schwarz M, Pugatch J, Augustson E (2015). Assessing electronic cigarette-related tweets for sentiment and content using supervised machine learning. J Med Internet Res.

[38] Kavuluru R, Sabbir A (2016). Toward automated e-cigarette surveillance: spotting e-cigarette proponents on Twitter. J Biomed Inform.

[39] Allem J, Ferrara E, Uppu SP, Cruz TB, Unger JB (2017). E-cigarette surveillance with social media data: social bots, emerging topics, and trends. JMIR Public Health Surveill.

[40] van der Tempel J, Noormohamed A, Schwartz R, Norman C, Malas M, Zawertailo L (2016). Vape, quit, tweet? Electronic cigarettes and smoking cessation on Twitter. Int J Public Health.

[41] Lazard AJ, Saffer AJ, Wilcox GB, Chung AD, Mackert MS, Bernhardt JM (2016). E-cigarette social media messages: a text mining analysis of marketing and consumer conversations on Twitter. JMIR Public Health Surveill.

[42] Cui K, Zheng X, Zeng D, Leischow S (2014). An Empirical Analysis on Communications about Electronic Nicotine Delivery Systems (ENDS) in Chinese Social Media. International Conference on Smart Health.

[43] Wen W, Zhang Z, Li Z, Liang J, Zhan Y, Zeng DD, Leischow SJ (2020). Public reactions to the cigarette control regulation on a Chinese microblogging platform: empirical analysis. J Med Internet Res.

[44] Askell-Williams HMJ (2004). A correspondence analysis of child-care students' and medical students' knowledge about teaching and learning. International Education Journal.

[45] Greenacre M (1992). Correspondence analysis in medical research. Stat Methods Med Res.

[46] Beishuizen JJ, Hof E, van Putten CM, Bouwmeester SJ, Asscher JJ (2001). Students' and teachers' cognitions about good teachers. Br J Educ Psychol.

[47] Tang L, Choi S, Morrison AM, Lehto XY (2009). The many faces of Macau: a correspondence analysis of the images communicated by online tourism information sources in English and Chinese. J Vacat Mark.

[48] Bischoff P (2014). A look inside Zhihu, China’s Answer to Quora. Techinasia.

[49] Shu C (2019). How Zhihu Has Become One of China's Biggest Hubs for Experts. Techcrunch.

[50] Dudarenok AG (2018). Econsultancy.

[51] Yang S (2019). Zhouyuan, Ceo of Zhihu: the Number of Users Has Exceeded 220 Million, Exploring Different Liquidation Paths. Sohu.

[52] Hoffman DL, Franke GR (1986). Correspondence analysis: graphical representation of categorical data in marketing research. J Mark Res.

[53] (2001). SPSS Categories Reference Guide. Eleventh Edition.

[54] Hair JF, Anderson RE, Tatham RL, Black WC (1998). Multivariate Data Analysis.

[55] Bendixen M (1996). A practical guide to the use of correspondence analysis in marketing research. Mark Res On-Line.

[56] Bock T How to Interpret Correspondence Analysis Plots (It Probably Isn’t the Way You Think). Displayr: Analysis and Reporting Software for Survey Data.

[57] Bock T How Correspondence Analysis Works (A Simple Explanation). https://www.displayr.com/how-correspondence-analysis-works/.

[58] Lyu J, Wang D, Mao Z, Ling P (2020). Evolution of Media Frames About E-cigarettes From 2004 to 2019: A Content Analysis of Newspapers in China. The 70th Annual Conference of International Communication Association.

[59] Liu Y (2006). 'Ruyan' Status Leads to Embarrassment of Supervision, Claiming to 'fill Legal Gap'. Beijing Times.

[60] Wan J (2018). The State Administration of Market Regulation and the State Tobacco Monopoly Administration Issued a Notice Banning the Sale of Electronic Cigarettes to Minors. Legal Daily.

[61] Goodchild M, Zheng R (2018). Early assessment of China’s 2015 tobacco tax increase. Bull World Health Organ.

[62] Sharpless N (2019). How FDA is Regulating E-Cigarettes. US Food and Drug Association.

[63] McCombs M, Shaw D (1972). The agenda-setting function of mass media. Public Opinion Q.

[64] Smith KA (2016). Effects of newspaper coverage on community issue concerns and local government evaluations. Communication Research.

[65] Chen M, Huang R (2018). Where has opinion leader gone? Analysis of the discussion of Hainan arbitration case on Weibo, Wechat and Zhihu. Shanghai J Rev.

